# Breast tissue imaging atlas using ultra-fast confocal microscopy to identify cancer lesions

**DOI:** 10.1007/s00428-024-03783-y

**Published:** 2024-03-19

**Authors:** Marie-Christine Mathieu, Moira Ragazzi, Malek Ferchiou, Paul J. van Diest, Odile Casiraghi, Aicha Ben Lakhdar, Nizar Labaied, Angelica Conversano, Muriel Abbaci

**Affiliations:** 1 Department of Medical Biology and Pathology, Gustave Roussy, Université Paris‐Saclay, Villejuif, France; 2 Surgery and Pathology Photonic Imaging Group, Gustave Roussy, Villejuif, France; 3 Pathology Unit, Azienda USL – IRCCS di Reggio Emilia, 42123 Reggio Emilia, Italy; 4 Dept. of Medical and Surgical Sciences for Children and Adults, University of Modena and Reggio Emilia, Modena, Italy; 5 Department of Pathology, University Medical Center Utrecht, 3584 CX Utrecht, The Netherlands; 6 SCM Bichat, 59 Rue Bichat, 75010 Paris, France; 7 Department of Breast and Plastic Surgery, Gustave Roussy, Université Paris‐Saclay, Villejuif, France; 8 UMS, AMMICa 23/3655, Plateforme Imagerie Et Cytométrie, Gustave Roussy, Université Paris-Saclay, Villejuif, France

**Keywords:** Ultra-fast fluorescence confocal microscopy, Ductal carcinoma in situ, Invasive carcinoma

## Abstract

New generation ultra-fast fluorescence confocal microscopy (UFCM) allows to image histological architecture of fresh breast tissue and may be used for ex vivo intraoperative analysis for margin status. The criteria to identify breast tumoral and non-tumoral tissues in UFCM images are still objects of investigation. The objective of the study was to create an atlas of ex vivo UFCM images of breast tissues and breast carcinomas based on the first extensive collection of large field-of-view UFCM breast images. One hundred sixty patients who underwent conserving surgery for breast cancer were included. Their fresh surgical specimens were sliced, stained with acridine orange, and imaged at high resolution with large-field-of-view UFCM. The resulting images were digitally false colored to resemble frozen sections. Each UFCM image was correlated with the corresponding definitive histology. Representative images of normal tissue, inflammation, benign lesions, invasive carcinoma (IC), and ductal carcinoma in situ (DCIS) were collected. A total of 320 large-field images were recorded from 58 IC of no special type, 44 invasive lobular carcinomas, 1 invasive mucinous carcinoma, 47 DCIS, 2 lobular carcinomas in situ, and 8 specimens without cancer. Representative images of the main components of the normal breast and the main types of ICs and DCIS were annotated to establish an UFCM atlas. UFCM enables the imaging of the fresh breast tissue sections. Main morphological criteria defined in traditional histopathology such as tissue architecture and cell features can be applied to describe UFCM images content. The generated atlas of the main normal or tumoral tissue features will support the adoption of this optical technology for the intraoperative examination of breast specimens in clinical practice as it can be used to train physicians on UFCM images and develop artificial intelligence algorithms. Further studies are needed to document rare breast lesions.

## Introduction

Breast cancer is the most prevalent cancer worldwide affecting women and stands as the leading cause of cancer-related deaths on a global scale [1]. Surgery is the main treatment option for early breast cancer and aims for a complete excision of the invasive and in situ carcinoma. The quality of the resection, which encompasses the complete removal of all cancerous tissue (free margin), is an important prognostic factor [2]. In case of breast conserving treatment, the surgeon needs to find the balance between removing the smallest possible breast lump containing the malignancy for optimal cosmesis and yet achieving free margins. This inevitably leads to a proportion of incomplete resections that may require re-excision; consequently, this incurs additional costs for the hospital or health systems and places an extra burden on the patient while negatively impacting cosmetic outcomes. Different imaging and microscopic methods have been applied for intraoperative examination of margins status [3], and although frozen sections and cytology offer the best sensitivity and specificity compared to radiography, ultrasonography, and other imaging methods, they are hampered by long turnaround times and the need for technician and pathologist resources [3]. As a consequence, the most commonly employed methods for intraoperative decision‐making on which surgeons still mainly rely is palpation and visual inspection, which are not sufficiently reliable in determining the margin status [4].

Optical methods based on light-tissue interactions have been proposed for clinical use in different cancer types [5, 6]. The miniaturization of optical components has boosted the emergence of new optical approaches for clinical implementation, such as confocal laser endomicroscopy [7, 8], optical coherence tomography [9], or photoacoustic imaging [10]. Confocal microscopy (CM) has been outlined as one of the most promising tools for morphological examination of fresh tissue in real time and with histological detail [11, 12]. Ex vivo CM allows fast digital imaging of unfixed tissue specimens. Two confocal imaging modalities were initially described, both creating grayscale images: (1) reflectance mode, based on the natural differences in refractive indices of subcellular structures within a given tissue; (2) fluorescence mode, based on the use of exogenous fluorophores to enhance morphology contrast. New generation ultra-fast fluorescence confocal microscopy (UFCM) devoted to ex vivo cell imaging of fresh tissue has been reported since a decade. It combines an ultra-fast scanning process with a large field-of-view of several square centimeters allowing intraoperative assessment and potentially rapid clinical decision-making [12–14]. The Histolog Scanner is an UFCM based on a 488-nm diode laser used in combination with Histolog Dip (acridine orange dye), providing high-resolution imaging of spatial cellular disposition similar to histopathology [12]*.* In breast, this UFCM has been recently described in three ex vivo studies presenting the tumoral versus non-tumoral interpretation of margins and tumor cores by pathologists and breast surgeons [15–17].

In one the abovementioned studies, we collected the first extensive data of UFCM breast cancer images during the HIBISCUSS (High resolution Imaging for Breast carcInoma detection in *ex-vivo* Specimens after breast Conserving sUrgery by hiStolog Scanner) study in which a training program and performance assessments were realized [17]. This study showed that UFCM images from invasive lobular carcinoma (ILC), invasive carcinoma of no special type (IC-NST), or ductal carcinoma in situ (DCIS) could be interpreted as tumoral by pathologists with a sensitivity of 98% and by surgeons with a sensitivity of 91%. No benign lesion, rare tumors, and radial scars were submitted to blind assessment.

Here, we present the created images database containing descriptions of different breast normal or tumoral tissues to provide an illustrated guide useful for physicians interested in the field.

## Materials and methods

### Patients

The study included 160 ex vivo human breast specimens from 160 patients that underwent primary conserving surgery at Gustave Roussy from June 2019 to January 2021. The patients were enrolled in the HIBISCUSS study, a prospective non-interventional preclinical single-arm study as already described [17]. ClinicalTrials.gov Identifier: NCT04976556.

Selected patients had a preoperative histological diagnosis of IC-NST, DCIS, or ILC.

The surgical procedure, imaging procedure, and imaging system have been previously described [17] and are briefly summarized below for the purpose of clarity.

### Surgical procedure

Breast surgery was scheduled and performed according to the standard of care at Gustave Roussy. Lumpectomy was orientated with physical marks following standard surgical procedures and immediately sent to the pathology department. A senior pathologist placed two single-use colored plastic clips on the upper and outer locations to maintain the anatomical orientation during specimen handling. The use of clips was mandatory as conventional inks interfere with fluorescence reading and could not be applied before UFCM imaging. The specimen was sliced following the frontal plane to produce two sub-specimens representing the superficial half and the deep half. Thicker specimens were cut into three parts following the frontal plane to produce superficial third, middle third, and deep third specimens. Intraoperative macroscopic and/or microscopic examination were performed before UFCM imaging if requested by the standard of care.

The specimens were dried with a surgical pad and stained for 10 s in the dedicated contrast agent Histolog Dip (SamanTree Medical) which is a specific solution of the acridine orange fluorescent dye. Acridine orange is a cell-permeant nucleic acid binding dye that emits green fluorescence when bound to DNA and RNA (λ excitation = 502 nm and λ emission = 525 nm). The specimens were then rinsed for 5 s in 0.9% NaCl solution and dried with a surgical pad.

Then, each specimen was placed over the imaging window. A surgical instrument could be gently applied onto the tissue to flatten out the imaged surface and optimize the imaging process. All sections of the lumpectomy specimen were scanned with UFCM (Histolog Scanner, SamanTree Medical, Switzerland).

The fluorescence images were generated as *en face* view and displayed with an artificial purple coloring of the grey values, resembling the result of a mono reagent coloration such as hematoxylin observed by pathologists in conventional frozen sections.

### Imaging system

The Histolog Scanner is an IVD CE-marked fluorescence confocal microscope designed for use in a clinical setting on large tissue specimens. The Histolog Scanner is housed in a compact setup: (0.76 m × 0.76 m x 1m56). It integrates a touch screen monitor to display the fluorescence confocal images and control device operation. The UFCM consists of a laser diode operating at 488 nm for fluorophore excitation combined with fluorescence signal collection above 500 nm. The confocal device can eliminate the “out-of-focus” signal from optical 0.5 × to digital 40 × magnifications. The acquired images correspond to a field of view of about 20 cm^2^ with a lateral resolution of up to 2 µm and an axial resolution of 30 µm. The images are acquired at a single depth of 20 µm below the tissue surface. The UFCM provides seamless images at once without additional image post-processing. UFCM builds a high-resolution image within 50 s independently of the sample size.

### Histopathology

After completion of UFCM imaging, each sample was fixed in 10% buffered formalin and underwent routine histopathology processing. Paraffin blocks were sectioned at 3 µm, and histological slides were stained by hematoxylin/eosin/saffron (HES).

Histological slides were then scanned at 20 × magnification with a NanoZoomer S210 Scanner (Hamamatsu Photonics, Massy, France) for data analysis and archiving.

### Atlas design

Final histopathological reports, location of tissue samples embedded in paraffin, and digital whole slide images of HES-stained sections were provided to three pathologists experienced in optical imaging (MR, MN, and PJvD) for analysis of UFCM images. The pathologists independently assessed the data using a dedicated viewer software and identified typical features in each UFCM image to highlight morphological interpretation criteria. All images were annotated to be exhaustive for tumoral components that can be recognized with UFCM. Finally, the most informative/representative images of normal or tumoral tissue were selected by two pathologists (MCM and MR) to publish the UFCM atlas.

The URL link for atlas website can be found in https://zenodo.org/records/10522358.

## Results

### Surgical specimen data

All 160 fresh surgical specimens were successfully imaged using UFCM. The duration for processing the breast specimen with UFCM image acquisition was 8 to 10 min. Physical integrity of all the specimens was preserved for our routine histopathology processes, and neither HES staining nor definitive histopathological analysis was impacted by the UFCM protocol. The final histological diagnosis was IC in 105 surgical specimens (58 IC-NST, 44 ILC, 1 mucinous carcinoma), 47 specimens showed DCIS. The higher proportion of IC-NST subtypes compared to ILC and DCIS corresponds to the usual rates of these lesions in the routine practice.

The final histological diagnosis of 10 specimens did not identify any cancerous lesions (IC or DCIS). In five cases, a large biopsy scar was identified suggesting a complete excision of the tumor by the preoperative biopsy. Two cases were diagnosed with lobular carcinoma in situ (LCIS). In three cases, lesion was not identified in the surgical specimens. One hundred percent of cases presenting tumor cells in definitive histology were also found by the pathologists at the time of correlation with UFCM images for atlas annotations.

### Morphological features and diagnosis criteria with UFCM

The UFCM images correspond to large digital histological sections of about 20 cm^2^, in which the pathologist can “navigate” and zoom in/out to review different levels of magnification. A total of 320 UFCM images were obtained and analyzed at multiple magnifications (from optical × 0.5 to digital × 40). The pathologist read the images at low magnification to analyze the architecture of the tissue. Then, regions of interest were assessed with higher magnification levels to identify tissue components.

The histological architecture in UFCM images is comparable to HES staining at low and intermediate magnifications. At high magnification, the nuclei appear dark-purple color in which hyperchromatism and large nucleoli can be identified from time to time. The cytoplasm of cells, collagen, and elastosis are light-purple color, whereas adipocyte cytoplasm appears in white color (unstained). Hypercellularity was observed by comparison with HES section, and it is probably due to 30 µm axial resolution of the device.

#### Normal breast tissue

**Fig. 1:**
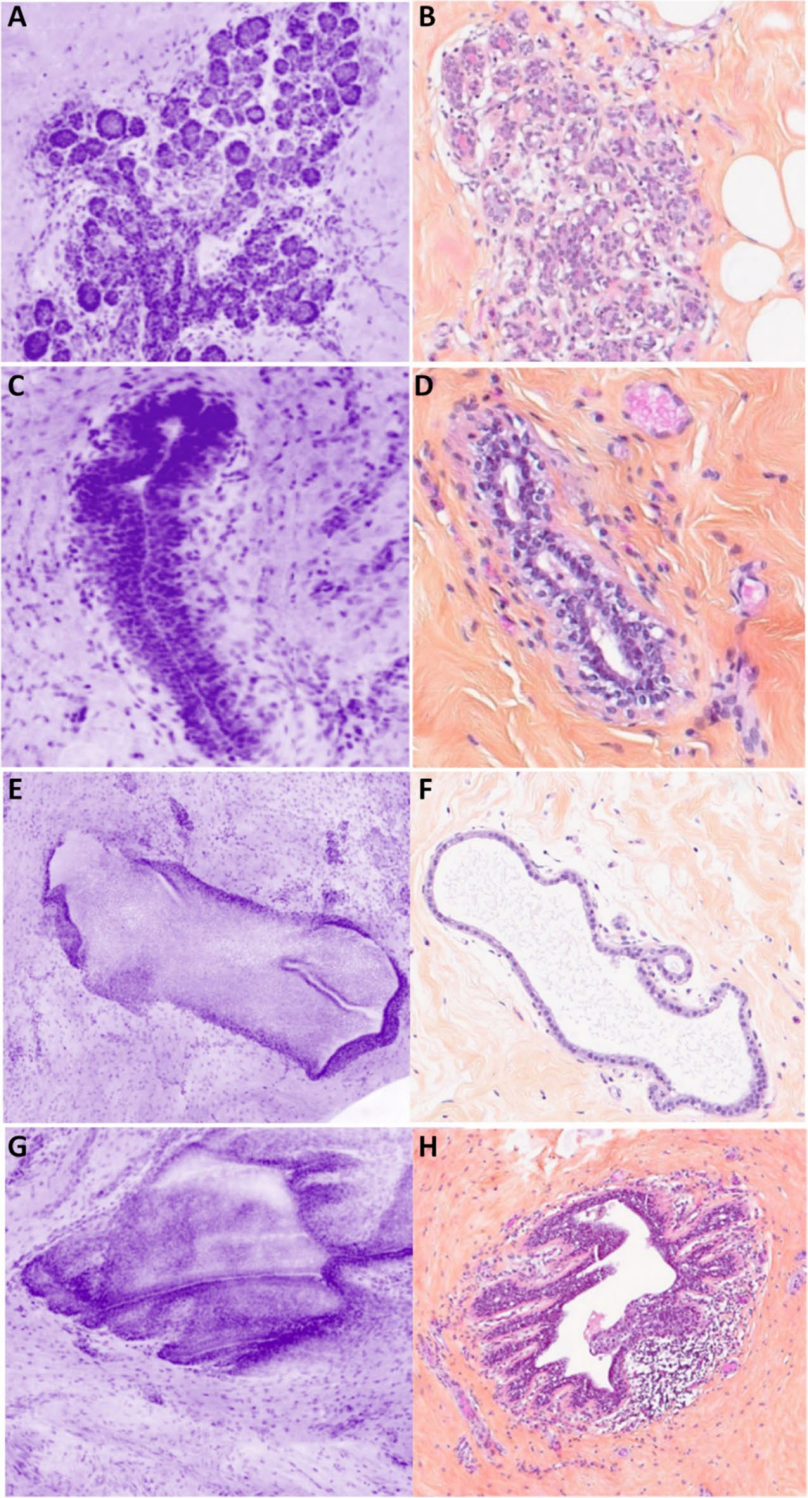
Normal epithelial structures. **A** Breast lobule seen as a bunch of grape-like structure, with a terminal duct ending in small and monotonous acini assembly. Stroma is slightly more cellular near the lobule (specialized stroma) (magnification zoom level 75%). **B** Corresponding HES section. **C** Longitudinal section of a normal duct presenting the typical pattern of more than two cell layers (magnification zoom level 100%). **D** Corresponding HES section. **E** Longitudinal section of a lactiferous duct (longitudinal section). The duct appears almost full of epithelial cells because of the 30-µm axial resolution and the orientation of the section. The honeycomb patterns is different from hyperplasia. A clearly defined lumen is always obvious (magnification zoom level 50%). **F** Corresponding HES section. **G** Lactiferous sinus (magnification zoom level 50%). **H** Corresponding HES section

**Fig. 2:**
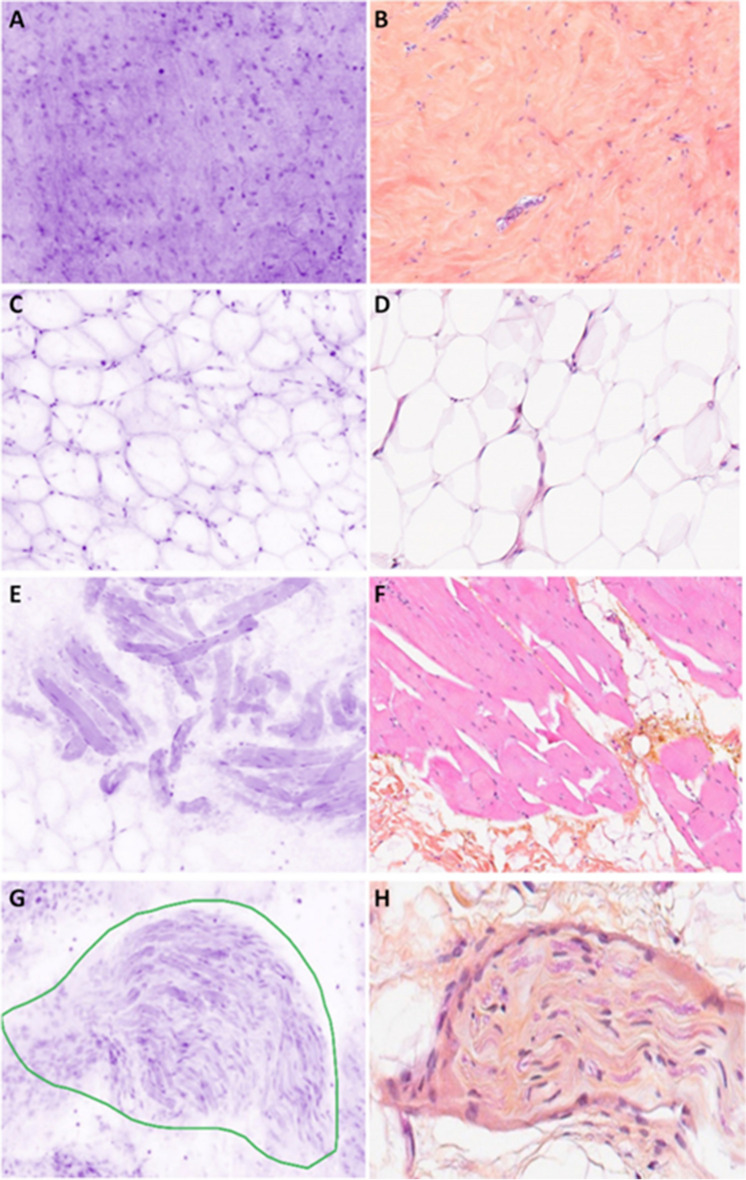
Normal connective tissue. **A** Fibrous hypocellular tissue (magnification zoom level 100%). **B** Corresponding HES section. **C** Adipose tissue (magnification zoom level 150%). **D** Corresponding HES section. **E** Skeletal muscle (magnification zoom level 50%). **F** Corresponding HES section. **G** Nerve encircled in green color (magnification zoom level 75%). **H** Corresponding HES section

Lobules were visible at low and high magnifications (Fig. 1A). Ducts are visualized with two different patterns: (1) a small cavity surrounded by two layers of cells (epithelial and myoepithelial) (Fig. 1C); (2) a duct filled by cells probably due to the 30-µm axial resolution (Fig. 1E). The cells had regular nuclei arranged in a honeycomb assembly which could be differentiated from a ductal proliferation (Fig. 1E).

**Fig. 3:**
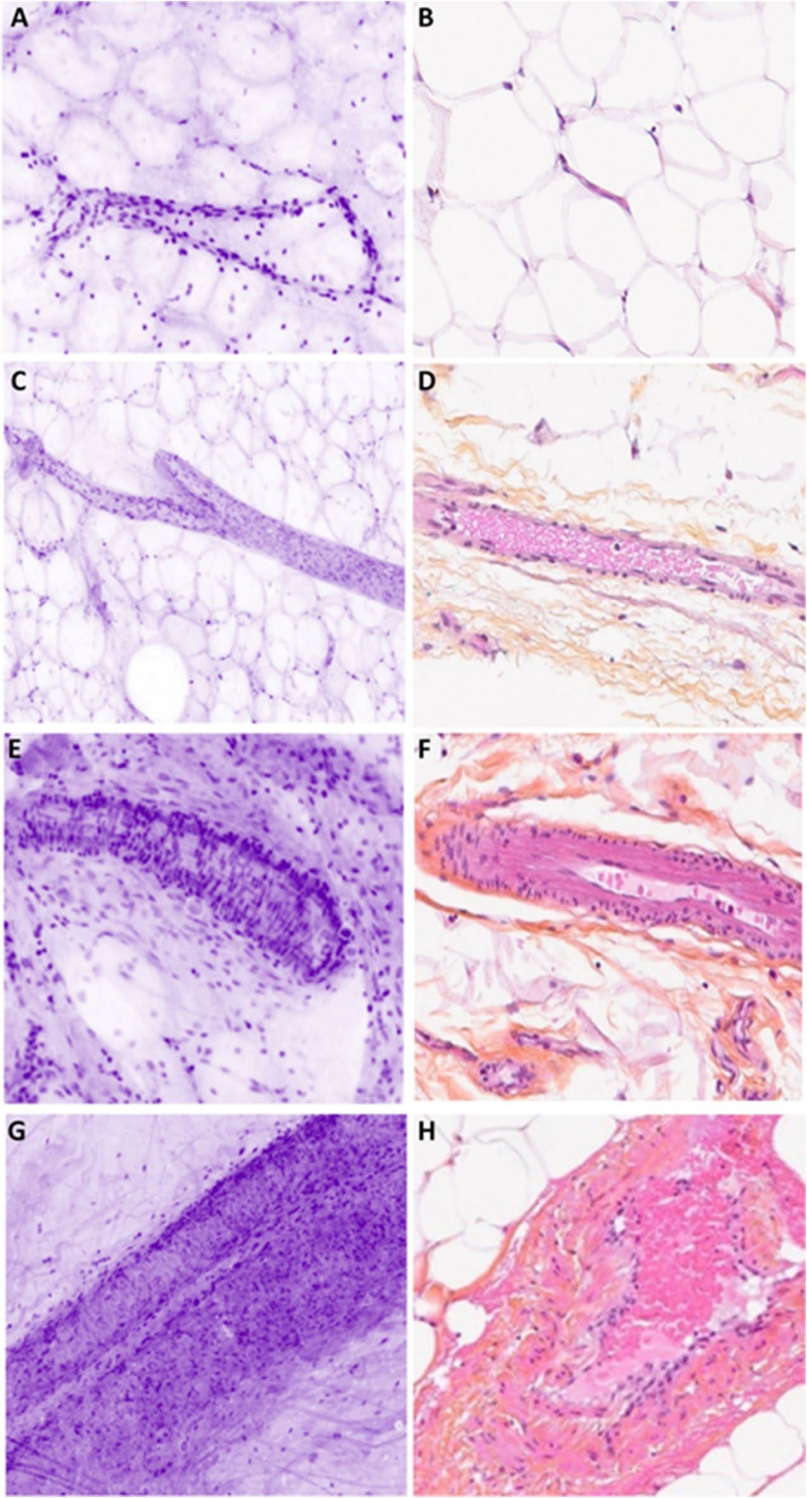
Vessels. **A** Capillaries in adipose tissue. The capillaries are seen in three dimensions and interconnected (magnification zoom level 150%). **B** Corresponding HES section. **C** Small vein, which branches in two venules with a thin three-layer wall (magnification zoom level 100%). **D** Corresponding HES section. **E** A small arteriole. Perpendicular lines from the muscular wall are helpful to differentiate vessels from normal ducts (magnification zoom level 100%). **F** Corresponding HES section. **G** Muscular arteriole with a thick wall and lumen in the middle. The wall is composed of three layers with different orientation of the muscular cells (magnification zoom level 100%). **H** Corresponding HES section

Connective tissue is identified by the shape of the fibers within the extracellular matrix (Fig. 2A). Adipocytes appears like large, round cells with an unstained cytoplasm limited by a thin membrane (Fig. 2C). Adipocytes, fibrous tissue, and capillary vessels are easy to diagnose and comparable in all cases (Figs. 2 and 3). Nerves are recognized with their well-defined structures constituted of parallel fusiform cells (Fig. 2G). Skeletal muscle is composed of multiple “cigar”-shaped cells (Fig. 2E). According to the axial resolution of 30 µm, the capillary vessel network is also easily identified (Fig. 3A–C). The larger vessels have a layer of muscular cells which has a characteristic perpendicular orientation on UFCM images (Fig. 3E–G).

#### Inflammatory features

**Fig. 4:**
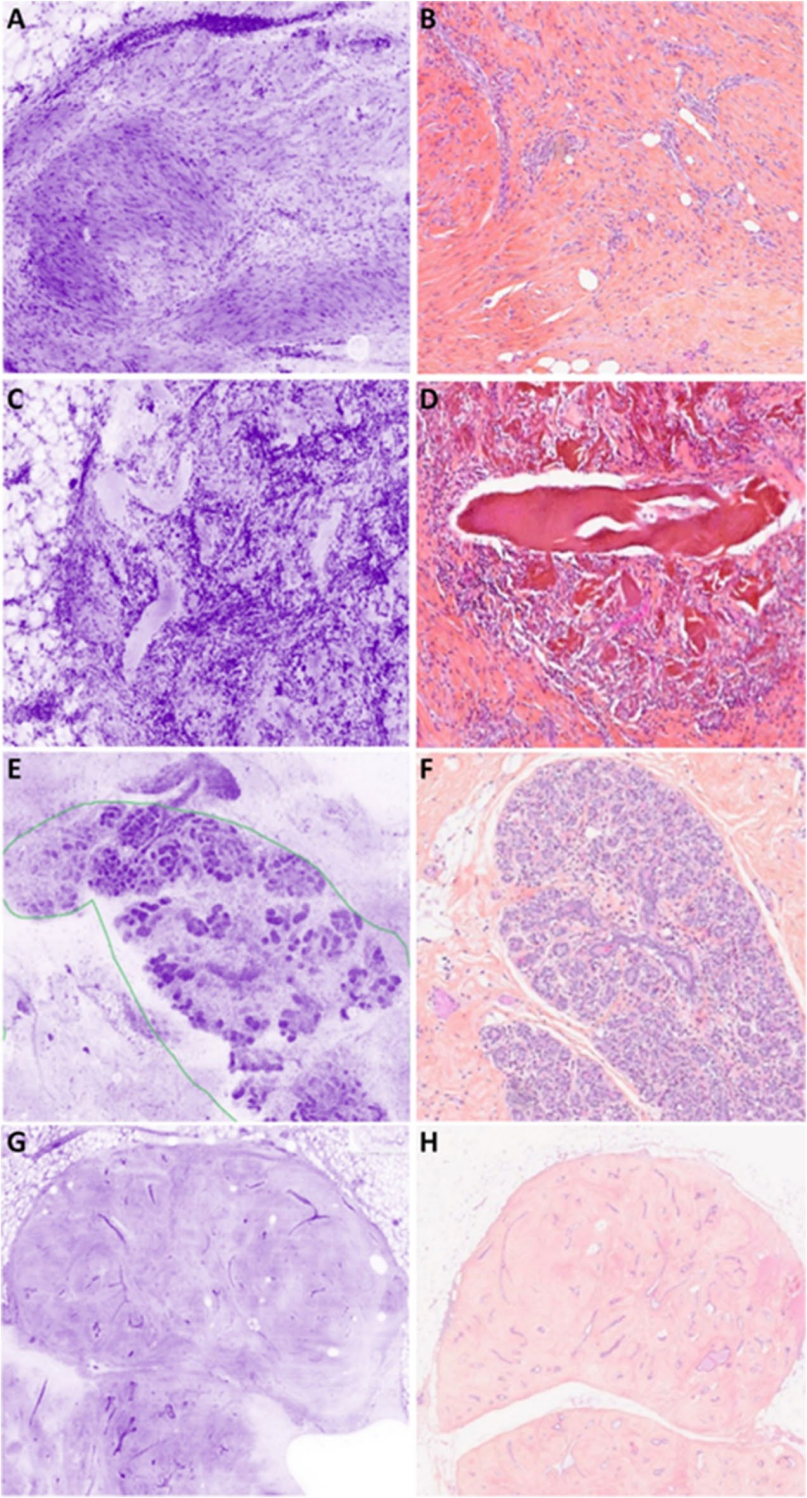
Benign lesions. **A** Scar of the biopsy (magnification zoom level 50%). **B** Corresponding HES section. **C** Inflammation around the exogenous collagen material placed after biopsy sampling (magnification zoom level 50%). **D** Corresponding HES section. **E** Adenosis encircled in green color (magnification zoom level 25%). **F** Corresponding HES section. **G** Fibroadenoma (magnification zoom level 25%). **H** Corresponding HES section

The fibrous scar of the preoperative biopsy and the associated lymphocyte infiltrates organized in aggregates are in general easily recognized (Fig. 4A). The collagen or mucin plug around the metallic tissue-marking clip can also be identified with confidence (Fig. 4C). However, the metallic clips seen at macroscopic examination are not visible by UFCM.

#### Benign lesions of the breast

Apocrine metaplasia is visualized as cells with large cuboidal cytoplasm and large nuclei. Fibroadenoma (Fig. 4G), fibrous mastopathy, and adenosis (Fig. 4E) have a typical architecture and are easily recognized at low magnification.

#### Carcinoma in situ

**Fig. 5:**
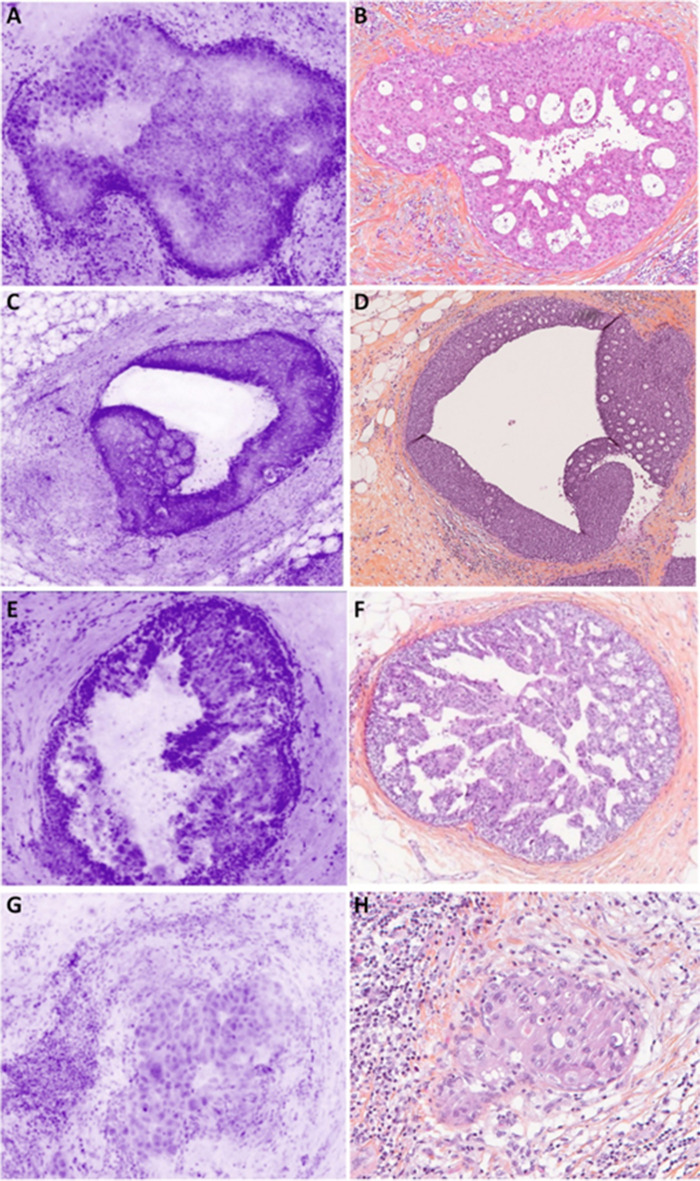
DCIS. **A** Low grade with cribriform architecture (magnification zoom level 50%). **B** Corresponding HES section. **C** Low grade (magnification zoom level 25%). **D** Corresponding HES section. **E** High grade (magnification zoom level 100%). **F** Corresponding HES section. **G** High grade with visible nucleoli and inflammatory infiltrate (magnification zoom level 100%). **H** Corresponding HES section

DCIS appeared as roundish, well-delimited areas of tumor cells larger than normal ducts or lobules (Fig. 5). The cribriform, solid, and papillary architecture can also be recognized. Their larger nuclei help to distinguish DCIS from normal epithelial cells of lobules or ducts. Sometimes, the presence of inflammation around neoplastic nests helps identify DCIS. However, neither necrosis nor microcalcifications are visible in UFCM images probably because they are not stained.

The diagnosis of classical LCIS is based on the distension of lobular unit with small and regular cells.

#### Invasive carcinoma

**Fig. 6:**
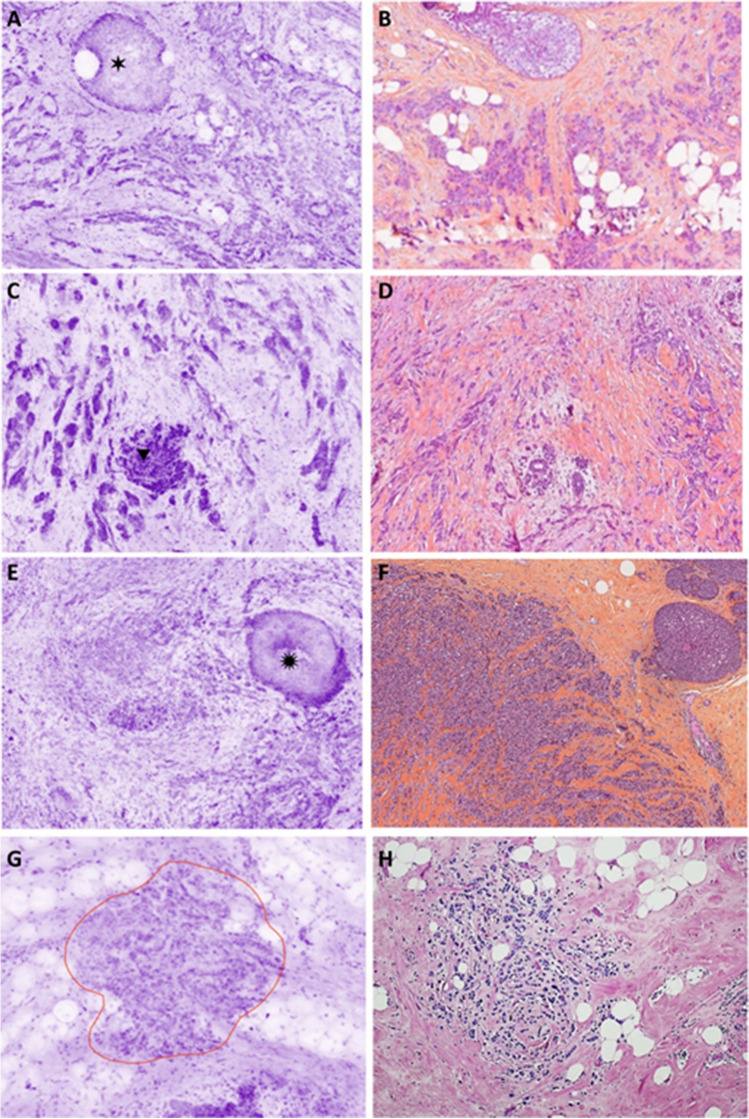
Invasive carcinoma. **A** NST with a low grade DCIS nest (six-pointed black star) (magnification zoom level 25%). **B** Corresponding HES section. **C** Low-grade NST. Tumor nests can be seen within connective tissue as well as an infiltrated normal lobule (upside-down triangle) (magnification zoom level 25%). **D** Corresponding HES section. **E** ILC with a high grade LCIS nest (twelve-pointed black star) (magnification zoom level 25%). **F** Corresponding HES section. **G** ILC encircled in red color. It is infiltrating fatty tissue (magnification zoom level 50%). **H** Corresponding HES section

Different levels of magnification are very useful for the diagnosis of IC (Fig. 6). The non-organoid organization of the lesion is seen at low magnification (Fig. 6A, E). When the stroma of the tumor was fibrous, the center of the carcinoma can be retracted and not in contact with the optical surface appearing as an empty hole.

The diagnosis of IC-NST (Fig. 6A–C) is based on the non-organoid organization of the tumor in tubules, solid nests, or trabeculae. It is also based on the density of the epithelial component and the size of the nuclei.

ILC (Fig. 6E–G) is identified with the presence of Indian file–arranged or single tumor cells encircling, in a targetoid fashion, pre-existing normal ducts or lobules. Some cases organized in solid nests are more difficult to distinguish from IC-NST. In several cases, the differential diagnosis from inflammation can be difficult due to the small size of the tumor cells.

Mucinous carcinoma is observed as small nests of carcinoma in mucin material with a slight staining.

#### Artifacts

**Fig. 7:**
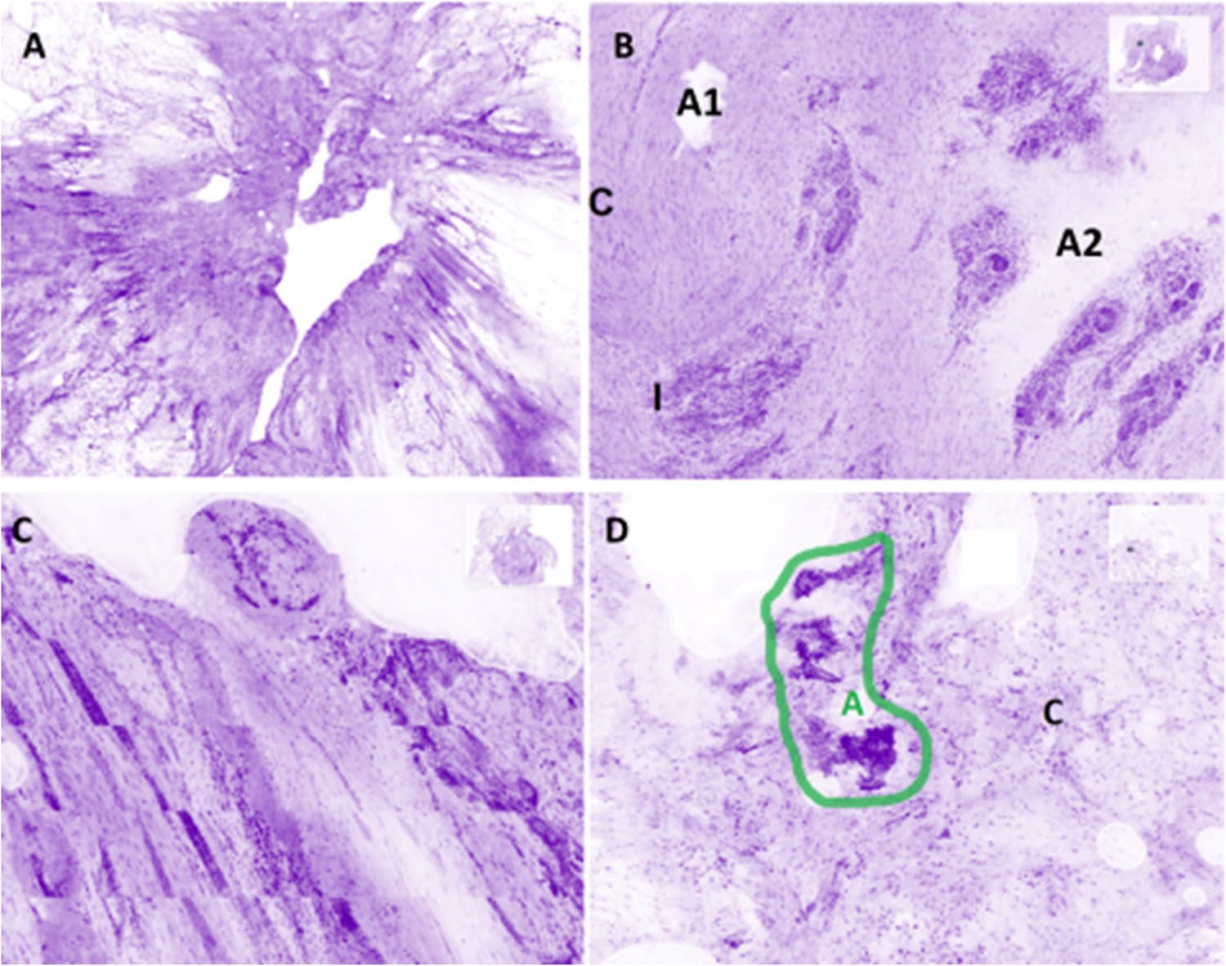
Typical artifacts in UFCM images. **A** Missing parts in the tumor core: they appear as “holes” in the images due to a lack of contact with the optical window (magnification zoom level 50%). **B** Artifacts which impeded tissue contact with the optical window. A1 is an impurity and the A2 area is presenting some fluid between the tissue and the optical window (magnification zoom level 25%). **C** Stitching effect due to the movement of the specimen during UFCM image acquisition (magnification zoom level 25%). **D** Tissue floaters overlaying the tissue specimen (encircled in green color) (magnification zoom level 25%). C, connective tissue; I, inflammatory cells; A, artifacts

Holes in the images represents the most frequent artifact. It is caused by tissue not in contact with the optical surface (Fig. 7A). Similarly, some fluid between the specimen and the interface could impact the visualization of the tissue (Fig. 7B).

A stitching effect is observed in some cases due to the movement of the specimen during the scanning process (Fig. 7C).

In rare cases, there are isolated groups of tumor cells that have been displaced during the slicing of the specimen. This “floater” phenomenon is similar to the tissue carryover that produces contamination in conventional histology. While potentially disturbing, this artifact could be recognized because these cells have no contact with the underlying tissue (Fig. 7D).

## Discussion

### UFCM imaging of breast normal and tumoral tissue

UFCM is a new method allowing the intraoperative examination of fresh tissue with limited processing. UFCM images provide a large field-of-view of surgical specimens (up to 20 cm^2^) allowing the simultaneous analysis of the main tumor and the surrounding breast tissue. The largest UFCM data collection from breast surgical specimens to date has been collected in the present study. None of the postoperative assessments showed artifacts that were attributed to UFCM imaging, indicating that UCFM is not tissue-traumatic as reported by Elfgen et al. [12].

The UFCM image allows the evaluation of the global architecture of the tissue at low magnification while higher magnifications (up to digital 40 ×) allow to explore and identify morphological features of the tumors. With an axial resolution of about 30 µm, UFCM images are richer than 3–4 µm histological sections in terms of cell density.

The different components of the normal tissue are usually recognized with confidence: adipocytes, fibroblasts, lobules, ducts, lymphocytes, nerves, and vessels. The adipose tissue is well preserved on fresh tissue with the UFCM technique and do not constitute an issue as observed in frozen sections. The 30-µm axial resolution provides a three-dimensional observation of vessels, ducts, and cysts which is confusing at the beginning and requires adequate training.

UFCM is providing lower level of magnification than conventional optical microscopes increasing the contribution of tissue architecture assessment in the diagnosis. The non-organoid organization of epithelial proliferations, the high density of cells, and stellar organization are typical of IC at low magnification. For IC-NST, at higher magnification, the architecture (cords or tubules), the size of the nuclei (compared to the size of the lymphocyte), and the stroma confirm the diagnosis.

The Indian files, the abundant stroma and the small size of tumor cells are characteristic of ILC visualized at high magnification. There are two difficulties for ILC: (1) the differential diagnosis with inflammation, especially in the scar of the preoperative biopsy. The size of the nuclei of ILC cells can be similar to activated lymphocytes; in these cases, the architecture of the lesion and the stromal organization may be of help for the diagnosis; (2) the differential diagnosis with IC-NST could also be difficult when ILC is not composed of dyscohesive cells and is presenting an architecture resembling to IC-NST, as previously shown [17]. These issues for the diagnosis of ILC can also be encountered in conventional histology, and this is usually addressed with E-cadherin immunohistochemical staining.

For DCIS, intraductal proliferation in dilated ducts could be easily recognized. The architecture of the proliferation is different compared to HES because of the 30-µm axial resolution of the microscope. The cribriform and the micropapillary pattern can be visualized but necessitate some training to be identified. High-grade DCIS has large nuclei, identifiable nucleoli, and lymphocytic infiltration around ducts. Low-grade DCIS may be difficult to differentiate from LCIS or atypical ductal hyperplasia. Microcalcifications and necrosis could not be seen on UFCM images. This phenomenon was likely due to the absence of fluorescence dye both in calcifications that lack nucleic acid for binding and in necrotic areas in which nucleic acids are highly degraded.

In the HIBISCUSS study, we selected patients with a pre-operative diagnosis of the most frequent breast carcinomas: IC-NST, DCIS, and ILC. The analysis of all lesions (tumoral or not) present in the UFCM images has been performed. However, some rarer IC subtypes and benign lesions, as well as tumor after neoadjuvant chemotherapy, are missing in the current atlas, and further studies addressing these topics are underway.

### UFCM image acquisition

The pseudo-color mode used in the UFCM images replaces the standard grayscale in CM. It resembles to histological sections that are more familiar to pathologists, and this facilitated the interpretation of the images [18, 19]. With an approximate axial resolution of 30 µm, UFCM images provide a light three-dimension visualization of the tissue surface, especially for ductal and lobular structures. UFCM also permitted to visualize the network of small normal vessels in three dimensions.

In most cases, the core tumor mass was not imaged on the UFCM images and presented as an empty hole of various dimensions surrounded by tumor cells. This was caused by the lack of contact between the center of tumor and the imaging interface during UFCM acquisition, likely due to the scirrhous fibrous stroma in the center of IC. To limit this artifact, we used a surgical instrument to gently flatten out the specimen along the imaging window. This artifact may be considered an indirect marker of IC with abundant fibrous stroma.

Among the artifacts seen on UFCM images, the main problem was the stitching effect due to specimen movement during acquisition. This problem was seen only after image generation, necessitating repeated image acquisition of the surgical specimen. It was caused by “slow spreading” of the surgical specimen until its stabilization. A newer version of the UFCM device with a prediction tool of moving artifacts has been recently released to support users in reducing the occurrence of this specific artifact.

### UFCM clinical implementation

In 2019, the committee of the College of American Pathologists focused for the first time on ex vivo microscopy applications, studying the potential advantages of CM over conventional histopathology [20]. UFCM recently joined the clinical market offering a larger field-of-view (20 cm^2^) and faster acquisition (less than 1 min per image) compared to other CM commercially available [14]. The histological interpretation of the UFCM images is close to the HES semiology and the implementation of the technique for intraoperative examination of specimens, especially margins, could be a streamlined process. However, an UFCM training is recommended for pathologists to make a histological diagnosis on UFCM images with confidence. Some issues related to both image interpretation and tissue handling should be regarded to avoid misdiagnosis. Recently, two clinical studies, with 50 and 40 patients respectively, reported margins assessment of lumpectomies with UFCM [15, 16]. The interpretation was devoted to marginally trained surgeons and a pathologist experienced in CM. They noticed a sensitivity from 30.7 to 33.3% and a specificity from 60.3 to 85.1% for surgeons and sensitivity from 43.8 to 53.8% and a specificity from 84.1 to 85.2% for the pathologist. Togawa et al. underlined that the low performance in their study was mainly due to artifacts caused by the use of electric surgical knives with high intensity and to a delay between surgery and image acquisition that caused excessive surface tissue drying. These issues could seriously impair image quality compromising further interpretation. In contrast, Sandor et al. used cold blades and scissors and had no heavy delays between surgical excision and UFCM imaging to produce good image quality and concluded that intraoperative reading of UFCM images by surgeon and pathologist could lead to a potential reduction of 30% and 58.3% of re-operation, respectively [15]. Additionally, the retrospective analysis conducted by an expert pathologist on UFCM images in Sandor et al. showed that as much as 75% of the re-operations could have been prevented with additional training on UFCM images. This finding underscores the need for adequate training material, and the present atlas as well as the learning program for pathologists and surgeons already developed in HIBISCUSS project [17] are believed to address this need. Finally, the creation of this large UFCM images database could be used to develop a deep learning model for image interpretation, which would constitute a supporting tool for decision-making of the clinician [21]. Due to the large field of view and false-coloring display of images, the atlas is currently specific to the Histolog Scanner but it could be used for other devices that would be developed in the future with the same approach*.*

## Conclusion

UFCM provides a quick assessment of microscopic architecture and content of breast tissues while preserving tissue integrity. Image quality is good enough to intraoperatively identify tumor tissue and major stromal components on large fresh tissue specimens. If validated in further clinical studies, UFCM could become a powerful tool for the assessment of lumpectomy specimens in an intraoperative setting. A training is highly recommended to pathologists and surgeons who start using this optical technique.

## References

[1] World Health Organization (2020) World cancer report: cancer research for cancer prevention. IARC

[2] Schnitt SJ, Moran MS, Houssami N, Morrow M (2015) The Society of Surgical Oncology-American Society for Radiation Oncology consensus guideline on margins for breast-conserving surgery with whole-breast irradiation in stages I and II invasive breast cancer: perspectives for pathologists. Arch Pathol Lab Med 139:575–577. 10.5858/ARPA.2014-0384-ED 25153620 10.5858/arpa.2014-0384-ED

[3] St John ER, Al-Khudairi R, Ashrafian H et al (2017) Diagnostic accuracy of intraoperative techniques for margin assessment in breast cancer surgery a meta-analysis. Ann Surg 265:300–310. 10.1097/SLA.0000000000001897 27429028 10.1097/SLA.0000000000001897

[4] Heidkamp J, Scholte M, Rosman C et al (2021) Novel imaging techniques for intraoperative margin assessment in surgical oncology: a systematic review. Int J Cancer 149:635. 10.1002/IJC.33570 33739453 10.1002/ijc.33570 PMC8252509

[5] Grosenick D, Bremer C (2020) Fluorescence imaging of breast tumors and gastrointestinal cancer. Recent Results Cancer Res 216:591–624. 10.1007/978-3-030-42618-7_18 32594400 10.1007/978-3-030-42618-7_18

[6] Tringale KR, Pang J, Nguyen QT (2018) Image-guided surgery in cancer: a strategy to reduce incidence of positive surgical margins. Wiley Interdiscip Rev Syst Biol Med 10:e1412. 10.1002/wsbm.1412 29474004 10.1002/wsbm.1412

[7] Abbaci M, Casiraghi O, Vergez S et al (2022) Diagnostic accuracy of in vivo early tumor imaging from probe-based confocal laser endomicroscopy versus histologic examination in head and neck squamous cell carcinoma. Clin Oral Investig 26:1823–1833. 10.1007/s00784-021-04156-4 34636941 10.1007/s00784-021-04156-4

[8] Chang TP, Leff DR, Shousha S et al (2015) Imaging breast cancer morphology using probe-based confocal laser endomicroscopy: towards a real-time intraoperative imaging tool for cavity scanning. Breast Cancer Res Treat 153:299–310. 10.1007/s10549-015-3543-8 26283299 10.1007/s10549-015-3543-8

[9] De Leeuw F, Abbaci M, Casiraghi O et al (2020) Value of full-field optical coherence tomography imaging for the histological assessment of head and neck cancer. Lasers Surg Med 52:768–778. 10.1002/lsm.23223 32072655 10.1002/lsm.23223

[10] Lin L, Wang LV (2021) Photoacoustic Imaging. Adv Exp Med Biol 3233:147–175. 10.1007/978-981-15-7627-0_8 34053027 10.1007/978-981-15-7627-0_8

[11] Longo C, Pampena R, Bombonato C et al (2019) Diagnostic accuracy of ex vivo fluorescence confocal microscopy in Mohs surgery of basal cell carcinomas: a prospective study on 753 margins. Br J Dermatol 180:1473–1480. 10.1111/bjd.17507 30512198 10.1111/bjd.17507

[12] Elfgen C, Papassotiropoulos B, Varga Z et al (2019) Comparative analysis of confocal microscopy on fresh breast core needle biopsies and conventional histology. Diagn Pathol 14:58. 10.1186/S13000-019-0835-Z 31202280 10.1186/s13000-019-0835-z PMC6570850

[13] Peters N, Schubert M, Metzler G et al (2018) Diagnostic accuracy of a new *ex vivo* confocal laser scanning microscope compared to H&E-stained paraffin slides for micrographic surgery of basal cell carcinoma. J Eur Acad Dermatology Venereol. 10.1111/jdv.15243 10.1111/jdv.15243 30198589

[14] Grizzetti L, Kuonen F (2022) Ex vivo confocal microscopy for surgical margin assessment: a histology-compared study on 109 specimens. Ski Heal Dis. 10.1002/ski2.91 10.1002/ski2.91 PMC9168011 35677928

[15] Sandor MF, Schwalbach B, Hofmann V et al (2022) Imaging of lumpectomy surface with large field-of-view confocal laser scanning microscope for intraoperative margin assessment - POLARHIS study. Breast 66:118–125. 10.1016/j.breast.2022.10.003 36240525 10.1016/j.breast.2022.10.003 PMC9574757

[16] Togawa R, Hederer J, Ragazzi M et al (2023) Imaging of lumpectomy surface with large field-of-view confocal laser scanning microscopy “Histolog® scanner” for breast margin assessment in comparison with conventional specimen radiography. Breast 68:194–200. 10.1016/J.BREAST.2023.02.010 36842192 10.1016/j.breast.2023.02.010 PMC9988675

[17] Conversano A, Abbaci M, van Diest P, Roulot A, Falco G, Ferchiou M, Coiro S, Richir M, Genolet PM, Clement C, Casiraghi O, Lahkdar AB, Labaied N, Ragazzi M, Mathieu MC (2023) Breast carcinoma detection in ex vivo fresh human breast surgical specimens using a fast slide-free confocal microscopy scanner: HIBISCUSS project. BJS Open. 7(3):zrad046. 10.1093/bjsopen/zrad046 10.1093/bjsopen/zrad046 PMC10182737 37178160

[18] Dobbs JL, Ding H, Benveniste AP et al (2013) Feasibility of confocal fluorescence microscopy for real-time evaluation of neoplasia in fresh human breast tissue. J Biomed Opt 18:106016. 10.1117/1.JBO.18.10.106016 24165742 10.1117/1.JBO.18.10.106016

[19] Ragazzi M, Piana S, Longo C et al (2014) Fluorescence confocal microscopy for pathologists. Mod Pathol 27:460–471. 10.1038/modpathol.2013.158 24030744 10.1038/modpathol.2013.158

[20] Mathur SC, Fitzmaurice M, Reder NP et al (2019) Development of functional requirements for ex vivo pathology applications of in vivo microscopy systems: a proposal from the In Vivo Microscopy Committee of the College of American Pathologists. Arch Pathol Lab Med 143:1052–1057. 10.5858/arpa.2018-0482-OA 30763117 10.5858/arpa.2018-0482-OA

[21] Combalia M, Garcia S, Malvehy J et al (2021) Deep learning automated pathology in ex vivo microscopy. Biomed Opt Express 12:3103. 10.1364/BOE.422168 34221648 10.1364/BOE.422168 PMC8221965

